# The patterns of facial fractures in traumatic brain injury (TBI) patients using ordinal regression: a retrospective study of five years

**DOI:** 10.3934/Neuroscience.2022019

**Published:** 2022-07-26

**Authors:** Mohamad Arif Awang Nawi, Nor Farid Mohd Noor, Ramizu Shaari, Ameera Kamal Khaleel, Muhamamd Amirul Mat Lazin, Ibrahim Mohammed Sulaiman, Mustafa Mamat

**Affiliations:** 1 School of Dental Sciences, Health Campus, Universiti Sains Malaysia, Kubang Kerian, 16150 Kota Bharu, Kelantan, Malaysia; 2 Faculty of Medicine, Universiti Sultan Zainal Abidin (UniSZA), Medical Campus, Jalan Sultan Mahmud, 20400 Kuala Terengganu, Terengganu Darul Iman, Malaysia; 3 Institute of Strategic Industrial Decision Modelling (ISIDM), School of Quantitative Sciences, Universiti Utara Malaysia, Sintok, 06010, Kedah, Malaysia; 4 Faculty of Informatics and Computing, Universiti Sultan Zainal Abidin, Terengganu, Malaysia

**Keywords:** traumatic brain injury, facial fractures, ordinal regression analysis

## Abstract

According to the World Health Organization (WHO), traumatic brain injury (TBI) will mainly contribute to disability and death by 2020. Facial fractures associated with TBI are a significant public health concern worldwide. The main etiological factors are road traffic accidents, violence, and falls. Neurological injury associated with facial fractures has been reported to be as high as 76%. Therefore, we retrospectively evaluated facial fracture patterns in patients with a traumatic brain injury in Hospital Universiti Sains Malaysia and evaluated their associations in our study. Ordinal regression was used to examine the facial fracture patterns in patients with traumatic brain injuries. The confounding variables were controlled using ordinal regression analysis, and probabilities of *p* < 0.1 were considered significant associations. The results found that zygomatic arch fracture −1.141 (95% CI, −2.487 to 0.204, p-value = 0.096), Le Fort II fracture −1.080 (95% CI, −2.138 to −0.022, p-value = 0.045), maxillary bone fracture 2.924 (95% CI, 1.784 to 4.063, p-value .001), nasal bone fracture 4.047 (95% CI, 1.243 to 6.851, p-value = 0.005), and mandibular bone fracture 1.501 (95% CI, 0.711 to 2.291, p-value .001) were the most common facial fracture types associated with traumatic brain injury (TBI). This study provides valuable data for creating prevention plans and gives a chance to discover the epidemiology, prevalence, and connection between TBI and facial fracture.

## 1. Introduction

Facial fractures and concurrent injury have been the focus of many investigations over the past four decades. Historically, facial architecture has been considered a cushion of impact, cushioning the neurocranium from severe damage [1]. According to the World Health Organization (WHO), in 2020, traumatic brain injury (TBI) will overtake several other ailments as the leading source of disability and death. The trauma of a brain injury can affect a person physically and psychologically. Traumatic brain injuries can disrupt brain function, affecting permanent or long-term physical, psychological, emotional, behavioral, and cognitive well-being. Injury to the brain is also a severe injury that needs immediate attention and is a significant public health problem [2].

For a person with TBI, there will be changes in cognitive function, such as impaired performance on daily tasks that require memory, language, spatial, or verbal abilities. TBI can be either temporarily or permanently. If TBI affects the motor centers in the brain, mobility may also be affected, and patients need mobility devices such as wheelchairs to assist with daily tasks. The objective way to determine structural brain damage after trauma is to use CT scanning. In addition, it is believed to indicate the severity of brain injuries and predict what will happen going forward. And it turns out that individual CT characteristics are crucial to predicting outcomes for TBI patients. Research on how cerebral lesions affect cranial pressure and death rates has varied dramatically in the scientific literature [3].

TBI can also affect a patient's behaviour causing changes in a person's personality. It is possible that, after the onset of TBI, a previously calm person may become impulsive or aggressive [3]. Facial fracture classification can affect multiple or single bones of the face. Because of the area's complexity, it is not always easy for the surgeon to classify facial fractures based on the bone involved [4]. Naso-orbital-ethmoid (NOE) fractures, Nasal fractures, orbital wall fractures, maxillary sinus fractures, Le Fort I fracture, Le Fort II fracture, and Le Fort III fracture as midface fractures. Orbital floor, Zygomatic molar complex, arch fracture as lateral midface fractures and symphysis, mandibular condyle, coronoid process, ascending ramus, angle, horizontal or body ramus, and alveolar process as mandibular fractures [5].

TBI associated with facial fractures is a significant public health concern worldwide [6]. Accidents of road traffic, abuse, and falls are the main etiological factors [7],[8]. Regarding etiology, frontal sinus fractures were the most common in Amsterdam, where mandibular fractures are considered the most frequent in tertiary trauma facilities [9]–[11]. The neurological injury incidence is related to facial fractures. Motor vehicle crashes, bicycle crashes, auto versus pedestrian crashes, and falls from heights >20 feet had nine times the torso and extremity injuries rates compared to assault and ground-level falls of injury patients [12]. According to a recent study, the exact relationships between different types of facial fractures and brain injuries have yet to be determined [13]–[15]. Therefore, in this study, we retrospectively evaluated the correlation between facial fractures in patients with a traumatic brain injury in Hospital Universiti Sains Malaysia and assessed their patterns based on the ordinal regression model.

## 2. Materials and methods

This study was conducted retrospectively at the Hospital Universiti Sains Malaysia. Patients with facial fractures admitted to the hospital on their first visit were included in this research. The study considered 282 respondents from the Medical Record Unit, Hospital Universiti Sains Malaysia, from July 2013 to June 2018. One research assistant uses administrative data from an electronic patient management program to extract retrospective data. Age, sex, patient status, occupation, traumatic brain injury, trauma timing, traumatic incidents, and facial fracture type variables were collected based on the patients' medical records, radiographic and clinical examinations, and case history. Traumatic incidents are categorized as firearm injuries, falling from high levels, road accidents, work-related injuries, sports, assault, pedestrians, and others. The inclusion and exclusion criteria as shown in Table 1 below.

**Table 1.** Inclusion and Exclusion Criteria.

Inclusiom Criteria	Exclusiom Criteria
i. medical history of subsequent patients treated at the USM hospital from July 2013 to June 2018	i. Missing documentation that made it impossible to determine gender, age, cause of injury.
ii. Coexistence of traumatic brain injury with facial fractures	

Traumatic brain injuries (TBI) are usually measured using the Glasgow coma score (GCS) scale. The GCS scale is employed to describe the extent of impaired consciousness in traumatic brain injury. This scale is easy to use and quick to score traumatic patients in an emergency. An acute traumatic brain injury can be classified as severe (GCS 3 to 8), moderate (GCS 9 to 12), and mild (GCS 13 to 15) based on the relationship between the GCS score and outcome. The SPSS software version 26 and excel software were used to conduct the statistics. The Spearman correlation is used to determine the strength and direction of the association between traumatic brain injury and patient's demographic variables such as sex, age, status of patients, occupation, timing of trauma, traumatic incidents and death and alive status. The facial fracture patterns in patients with traumatic brain injuries were assessed. Their associations were examined using the odds ratio (OR) and 95 percent confidence interval (CI) using Ordinal Regression analysis. Ordinal regression was used to analyse the facial fracture patterns in patients with a catastrophic brain injury. The confounding variables were controlled using ordinal regression analysis, and *p*-value of less than 0.1 was considered statistically significant.

## 3. Results

### 3.1. Patients Demographic

The total number of individuals admitted to the USM hospital with facial fractures from 2015 to 2019 was 282, as considered in this study. In addition, the patient demographics profile revealed the distribution of facial fractures according to sex, representing 64 female and 218 male patients, with men outnumbering women with a ratio of 3:1. Most patients with facial fractures were between the ages of 11 to 20 (n = 110, 39%). The second higher age group was 21–30 years, with 76 (27%) patients. As shown in Table 1 below, the highest number of patients with facial fractures was the single status with 67.7% compared to married (31.2%), divorced (0.4%), and others status (0.7%). The majority of patients were students with 35.6%, the second-highest percentage was not stated as patients' occupation, and the lowest percentage was unemployed and retired with 3.6%. Most traumatic brain injury patients have an accidental peak around 6.00 pm to 11.59 pm compared to other times. The most severe traumatic brain injuries were mild traumatic brain injuries with 78.4% (n = 221), followed by severe traumatic brain injuries with 13.1% (n = 37), and then moderate traumatic brain injuries with 8.5% (n = 24). The most common traumatic incidents were motorcycle versus other accidents, accounting for 50.7% (n = 143), followed by motorcycle versus motorcycle accident 20.6% (n = 58), motor vehicle accidents 18.1% (n = 51), falling 4.3% (n = 12), domestic/home injury 1.8% (n = 5), pedestrian 1.4% (n = 4), assault 1.1% (n = 3), bicycle rider 0.7% (n = 2), sport, firearm injury, work-related injury and others 0.4% (n = 1). Majority of the patients with facial fractures were alive (n = 276 patients, 98%) and dead only n = 6, 2% patients.

**Table 2.** Patients Demographic Characteristics.

Variable	Frequency	Percentage
Age		
1-10	9	3.2
11-20	110	39.0
21-30	76	27.0
31-40	31	11.0
41-50	26	9.2
> 50	30	10.6
Sex		
Male	218	77.3
Female	64	22.7
Status of patients		
Single	191	67.7
Married	88	31.2
Divorced	1	0.4
Others	2	0.7
Occupation of patients		
Government	15	5.3
Private	12	4.3
Self-employment	54	19.1
Student	100	35.6
Unemployed	10	3.6
Retired	10	3.6
Not stated	80	28.4
Traumatic Head Injury		
Mild	221	78.4
Moderate	24	8.5
Severe	37	13.1
Timing of trauma		
0	28	9.9
0600-1159	58	20.6
1200-1759	90	31.9
1800-2359	105	37.2
Traumatic incidents		
Motor vehicle accident	51	18.1
Motorcycle vs motorcycle accident	58	20.6
Motorcycle vs other	143	50.7
Bicycle rider	2	0.7
Pedestrian	4	1.4
Fall	12	4.3
Assault	3	1.1
Sport	1	0.4
Firearm injuries	1	0.4
Work related injuries	1	0.4
Domestic/Home	5	1.8
Others	1	0.4
TBI Status of Patients		
Alive	276	98
Dead	6	2

### 3.2. Spearman Correlation Method

Table 3 below shows the Spearman correlation to measure the strength and direction of association between traumatic brain injury and patient demographic variables.

**Table 3.** Results of the Spearman Correlation analyses between Traumatic Brain Injury and Patients Demographics (N = 282).

Variables		TBI	Age Group	Sex	Status of Patient	Occupation Status	Timing of Trauma	Traumatic Incidents	Died or Alive
TBI	Correlation Coefficient	1.000	.032	-.136*	.013	-.119*	-.007	-.129*	.077
	Sig. (2-tailed)	.	.594	.022	.827	.047	.906	.030	.198
Age Group	Correlation Coefficient	.032	1.000	.065	.691**	-.010	-.184**	-.069	-.070
	Sig. (2-tailed)	.594	.	.279	.000	.867	.002	.251	.240
Sex	Correlation Coefficient	-.136*	.065	1.000	.151*	.081	-.048	.022	-.155**
	Sig. (2-tailed)	.022	.279	.	.011	.173	.425	.708	.009
Status of Patient	Correlation Coefficient	.013	.691**	.151*	1.000	-.169**	-.098	-.020	-.054
	Sig. (2-tailed)	.827	.000	.011	.	.004	.102	.735	.365
Occupation Status	Correlation Coefficient	-.119*	-.010	.081	-.169**	1.000	-.087	.150*	-.107
	Sig. (2-tailed)	.047	.867	.173	.004	.	.149	.012	.074
Timing of Trauma	Correlation Coefficient	-.007	-.184**	-.048	-.098	-.087	1.000	-.089	-.168**
	Sig. (2-tailed)	.906	.002	.425	.102	.149	.	.138	.005
Traumatic Incidents	Correlation Coefficient	-.129*	-.069	.022	-.020	.150*	-.089	1.000	.065
	Sig. (2-tailed)	.030	.251	.708	.735	.012	.138	.	.278
Died or Alive	Correlation Coefficient	.077	-.070	-.155**	-.054	-.107	-.168**	.065	1.000
	Sig. (2-tailed)	.198	.240	.009	.365	.074	.005	.278	.
GCS Score	Correlation Coefficient	1.000**	.032	-.136	.013	-.119	-.007	.073	.077
	Sig. (2-tailed)	.000	.594	.022	.827	.047	.906	.260	.198

*Correlation is significant at the 0.05 level (2-tailed).

**Correlation is significant at the 0.01 level (2-tailed).

*Statistical Analysis: Spearman's-rho analysis*

From the Table 3 above, traumatic brain injury has a very weak, negative correlation with sex (rs = −0.136, p = 0.022), occupation status (rs = −0.119, p = 0.047) and traumatic incident (rs = −0.129, p = 0.03). There was a strong, positive correlation between age and status of patients, which was statistically significant (rs = .691, *p* ≤ 0.001). Age has a weak correlation with the timing of trauma (rs = −0.184, p = 0.02). Besides that, sex has a very weak correlation with the status of patients (rs = 0.151, p = 0.011) and died or alive variable (rs = −0.155, p = 0.009). In addition, the status of patients has a very weak, negative correlation with occupation status (rs = −0.169, p = 0.004). Occupation status has a very weak correlation with traumatic incidents (rs = 0.150, p = 0.012). Next, the timing of trauma has a very weak and negative correlation with the died or alive variables (rs = −0.168, p = 0.005). There was a strong, positive correlation between traumatic brain injury and GCS score of patients, which was statistically significant (rs = 1.000, *p* ≤ 0.001). This mean that, the mild traumatic brain injury patients (GCS 13–15) reported over 74.8% survival, few with minor consequences.

### 3.3. Ordinal Regression Method

The chi-square difference has a significant level of less than 0.05, indicating the model's superiority with predictors over that without predictors (Table 4). Based on the goodness-of-fit model, the model fits well where the significant level is more than 0.05 (Table 5).

**Table 4.** Model Fitting Information.

Model	-2 Log Likelihood	Chi-Square	df	Sig.
Intercept Only	161.835			
Final	121.440	40.395	9	≤ 0.001

**Table 5.** Goodness of Fit Model.

	Chi-Square	df	Sig.
Pearson	68.132	81	.845
Deviance	66.065	81	.885

From the Table 6, zygomatic arch fracture −1.141 (95% CI, -2.487 to 0.204, p-value = 0.096), Le Fort II fracture −1.080 (95% CI, −2.138 to −0.022, p-value = 0.045), maxillary bone fracture 2.924 (95% CI, 1.784 to 4.063, p-value ≤ 0.001), nasal bone fracture 4.047 (95% CI, 1.243 to 6.851, p-value = 0.005), and mandibular bone fracture 1.501 (95% CI, 0.711 to 2.291, p-value ≤ 0.001) represent the most common type of facial fracture related to traumatic brain injury (TBI).

**Table 6.** Parameter Estimation of Facial Fracture in Traumatic Brain Injury (TBI).

	Estimate	Std. Error	Wald	df	Sig.	95% Confidence Interval	
						Lower Bound	Upper Bound
TBI = 1	3.701	.528	49.158	1	≤ 0.001	2.667	4.736
TBI = 2	4.393	.552	63.253	1	≤ 0.001	3.310	5.476
Zygomatic Complex Fracture=1	-.254	.366	.481	1	.488	-.970	.463
Zygomatic Complex Fractures=2	0	.	.	0	.	.	.
Zygomatic Arch Fractures=1	-1.141	.686	2.765	1	.096	-2.487	.204
Zygomatic Arch Fractures=2	0	.	.	0	.	.	.
Le Fort I fracture=1	.251	.507	.246	1	.620	-.742	1.245
Le Fort I fracture=2	0	.	.	0	.	.	.
Le Fort II fracture=1	-1.080	.540	4.005	1	.045	-2.138	-.022
Le Fort II fracture=2	0	.	.	0	.	.	.
Le Fort III fracture=1	-.387	.696	.309	1	.578	-1.751	.977
Le Fort III fracture=2	0	.	.	0	.	.	.
Maxillary Bone fracture=1	2.924	.581	25.298	1	≤ 0.001	1.784	4.063
Maxillary Bone fracture=2	0	.	.	0	.	.	.
Nasal Bone fracture=1	4.047	1.431	8.004	1	.005	1.243	6.851
Nasal Bone fracture=2	0	.	.	0	.	.	.
Mandibular Bone fracture=1	1.501	.403	13.871	1	≤ 0.001	.711	2.291
Mandibular Bone fracture=2	0	.	.	0	.	.	.
Orbital Wall fracture=1	-.305	.373	.668	1	.414	-1.037	.426
Orbital Wall fracture=2	0	.	.	0	.	.	.

## 4. Discussion

Abosadegh [16] supported this result where the young adult males aged 20–39 years represent the most persistent TBI related to the maxillofacial fracture (MFF). Incidents that cause traumatic brain injury were road accidents, assault, falls, and other causes, representing a lower percentage. The types of MFF (mandible, maxilla, zygomatic, and orbital) are linked to TBI in MFF patients. From the bounds of the TBI studied with the literature on the MFF, it is concluded that sex groups, age, adult culture, civilization, assault, and RTA are the significant features determining the tendencies of TBI-related TBFF.

Individuals with complex midface fractures are 57% (Relative risk = 1.57; P = 0.005) very likely to die than patients with simple midface fractures, according to Bellamy et al. [17]. When the complex midface fracture group was stratified by fracture pattern, they discovered Le Fort II fractures were primarily responsible for the association's strength. When examined separately, this was the only pattern that remained significantly predictive. Le Fort II fractures, in particular, raised the death risk by 94% (Relative risk = 1.94; P = 0.01), although Le Fort I and III fractures were not. Patients with Le Fort II were 2.88 times (p < 0.01) and Le Fort III fractures 2.54 times (p < 0.001) expected to have underlying intracranial injuries among patients without neurological disorders, respectively. Lucke-Wold et al [18]. supports the results in the study and states that Lefort type 2 is associated with severe injuries and can increase mortality in patients.

The study of Abosadegh et al. [6] showed similar results. They discovered a strong correlation between the GCS and the existence of TBI concurrent MFF. According to the logistic regression analysis, the presence of TBI was statistically related to individuals 31–40 years of age, the root of injury (RTA-traffic accident), all forms of midface fractures, and most kinds of mandibular fractures. In addition, statistical analysis using the logistic regression analysis method showed a correlation between TBI in MFF patients, RTA and MFF kinds which is the alveolar process of jaw fracture, maxillary sinus walls, orbital wall, zygomatic curve, zygomatic complex, and nasal bone.

In Turkey, according to Arslan et al. [9] study, 8.9% of people having MF trauma had TBI who are 18–39 years old, 73.7 percent were males, and 26.3 percent were females. Arslan et al. [9] showed that the main causes of frequent TBI were violence in cases with MF, trauma (47.8%), falls in high places (28.1%) and RTA (20.9%). About 33 affected individuals had mild brain injuries (GCS score: 13–15), 18 people with moderate brain injuries (GCS score: 9–12), and 17 persons with severe brain injuries (GCS score: 3–8). The Maxillary bone fractures represent 28% of bladder fractures, nasal fractures (25.3%), zygomatic fractures (20.2%), mandibular fractures (8.4%), and NOE fractures (3.1%) [9].

Salentijn et al. [8] conducted a retrospective investigation examining MF and TBI injury in Amsterdam and showed similar results. Over ten years, TBI was found in 8.1 percent of 579 patients with MFF. Almost 89.4% of those affected are men, with women representing just 10.6%, and the main age group for both sexes being 20–29 years. A road traffic accident is also the most common root of TBI-related mortality (55.3%). Falls represent 25.5%, violence accounts for about 4.3%, and the remaining represent only 14.9 percent. The frequent site of MFF (21.9%) is Frontal sinus fractures, and they discovered that 57.4% of TBI individuals had severe brain injuries, while 21.3 percent had mild to moderate injuries [8].

According to Zandi & Seyed Hoseini [19], a prospective study found that the brain injury rate connected with facial trauma is 23.3%. Furthermore, male patients with facial injuries had the highest brain injury rate (88.7%), whereas female patients had 11.3 percent. The most common causes of injury were motorcycle accidents (43.7%), motor vehicle accidents (29.8%), and assault (16.9%), with nasal bone fractures being the most common (45%), then mandibular bone fractures (36.4%), zygomatic bone fractures (26.8%), and Le Fort II (22.2%). According to their research, they found higher MCA association (211.30 times), MVA (139.43 times), falls (65.9 times), attacks (69.28 times), frontal sinus fracture (84.5 times), and Le Fort II TBI fracture sustainable.

According to Goil et al. [20], there is a relationship between traumatic brain injury (TBI) and maxillary fracture (MF) trauma in India. They reported that 81.3% of patients with MF injury were found to have concomitant TBI, were predominantly male, and were mostly between the ages of 20 to 40. The main recorded root of TBI in individuals having MF trauma was a road traffic accident (92.4%), fall from height (4.16%), and stroke (2.3%). In addition, zygomatic fractures significantly impacted TBI with an odds ratio of 3.34. Besides that, mandible, NOE, maxilla, and supraorbital bone also significantly impacted TBI with odds ratios of 2.46, 1.67, 1.36, and 1.15.

Our study confirms that Road Traffic Injury (RTI) is a significant public health concern as it is responsible for increasing traumatic brain injury. The primary cause of the trauma is high-impact traffic accidents, which disproportionately affect men. Males have a higher rate of traffic accidents due to their high activity levels and participation in high-risk activities such as aggressive driving or riding and excessive speeding without protective gear. Indeed, most patients in our study were motorcycle riders (50.7%). Furthermore, because women do not ride motorbikes or drive cars as frequently as men, the low number of female patients (22.7) can be explained. Over speed and poor conditions can explain the injury severity on the road or in the vehicle (failure of drivers and passengers to use helmets or seatbelts). The reasons listed above may also partially explain this study's high frequency of traffic accidents as the principal cause of a severe brain injury.

According to the intensity of the traumatic brain injury, this study was supported by Ansari study [21]. He stated that mild traumatic brain injury is the most prevalent, the second is severe, and the last is moderate traumatic brain injury. Kishanrao and Kishanrao [22] state that, the minor head injury patients with a GCS score between 13 to 15 reported over 90% having survival, only a few with minor consequences. And Patients are allowed to discharge directly from the ED and are often expected to have a better recovery than patients requiring hospitalization after mild TBI. In addition, road traffic accidents represent the leading cause of mild traumatic brain injury with maxillofacial fractures in 39.8% of patients. On the other hand, 14% of patients were associated with severe traumatic brain injuries [23]. Traumatic brain injuries (TBI) frequently result in double epidural hematomas (EDHs), with a mortality rate of more than 30 percent. Patients with double EDHs and a Glasgow Coma Scale (GCS) score between 3 and 8 have a 47.6 percent mortality rate, compared with a 25 percent mortality rate in patients with single EDHs and the same GCS score. Double EDHs result from a severe traumatic brain injury (TBI), which has a distinct presence and is associated with a high rate of morbidity and mortality [24]. According to Esmer et al. [25], about 25% of all severely injured patients have maxillofacial injuries. According to Pietzka et al. [26], an assault was the third-highest cause of maxillofacial injury. The nasal bone, mandible, zygomatic, and midface are the most often fractured bones following an assault [24].

## 5. Conclusions

The findings of this retrospective analysis provide critical information for the future design of injury prevention strategies. The most common causes of death in this retrospective research of 282 patients at Hospital Universiti Sains Malaysia were road traffic accidents (motorcycle vs. motorcycle accidents). Mild traumatic brain injury is the most prevalent type, and most bone fractures occur between the ages of 11 and 30 years. Zygomatic arch fractures, Le Fort II fractures, maxillary bone fractures, nasal bone fractures, and mandibular bone fractures are common in patients with severe brain injuries.

Based on this study, the higher traumatic brain injuries were mild with 78.4% and, therefore, a slight mortality rate of only two percent compared to 98 percent of surviving patients. However, these death cases due to non-surviving facial fractures have a dramatic tendency for middle and upper facial fracture patterns and death from neurological injury. Most importantly, patients with this fracture pattern must be carefully observed by the surgeon treating them.

Traumatic brain injuries (TBI) such as zygomatic arch fractures, Le Fort II fractures, nasal fractures, upper jaw fractures, or mandibular fractures should be taken seriously. The following facial fractures can contribute to the severity of traumatic brain injuries. And this study contributes to the patterns of facial fractures related to traumatic brain injuries for the development of prevention plans; it also provides an opportunity to investigate the epidemiology, prevalence, and connection of TBI and a facial fracture. Citizen awareness programs should be launched in combination with legislation requiring all citizens to adhere to preventive measures such as complying with traffic rules, organizing regulations requiring the use of helmets, avoiding dangerous driving, and improving road conditions.

## 6. Limitations

There were several limitations to this study. Malaysia is made up of 13 states, but only one of them, Kelantan, contributed studies to this analysis. Most of the articles reviewed had small patient populations, which provides a less accurate picture of a state with a population as large as Kelantan. On top of that, there is a disparity between rural and urban health care and population. This study only included studies from urban areas. As a result, while these findings shed some light on the potential contribution of brain injury to outcomes, more research is needed to understand better how these and other factors, such as preinjury risk factors and emotional trauma resulting from injury, affect recovery from traumatic injuries. Although our main outcome may be affected by a bias in the response, we investigated the correlation between facial fractures in traumatic brain injury patients. We assessed the fractures' patterns, thus strengthening our results. Unfortunately, we also do not have any data on patients who have been discharged, so it is impossible to determine the long-term consequences of traumatic brain injury (TBI) in those patients, as well as the actual mortality rate.

Other variables such as biomarkers, preinjury factors, multiple trauma, and post-injury social and rehabilitative support should be considered in future research to better understand. Besides, clinical decisions for individual patients should be informed by variables other than head CT scan status. In addition, future research needs to include data from several states in Malaysia to understand traumatic brain injury better. Finally, there is a need for additional research using TBI registries, which could be expanded to include all TBI patients and evaluate long-term outcomes to better understand the condition.

## References

[1] Kelamis JA, Mundinger GS, Feiner JM (2011). Isolated Bilateral Zygomatic Arch Fractures of the Facial Skeleton Are Associated with Skull Base Fractures. Plast Reconstr Surg.

[2] Hyder AA, Wunderlich CA, Puvanachandra P (2007). The impact of traumatic brain injuries: a global perspective. NeuroRehabilitation.

[3] Dawodu SD (2021). Traumatic Brain Injury (TBI) - Definition, Epidemiology, Pathophysiology: Overview, Epidemiology, Primary Injury. Phys Med Reh.

[4] Avery LL, Susarla SM, Novelline RA (2011). Multidetector and three-dimensional CT evaluation of the patient with maxillofacial injury. Radiol Clin North Am.

[5] Mihailova H (2006). CLASSIFICATIONS OF MANDIBULAR FRACTURES REVIEW. J IMAB.

[6] Abosadegh MM, Rahman SA, Saddki N (2017). Association of traumatic head injuries and maxillofacial fractures: A retrospective study. Dent Traumatol.

[7] Park KP, Lim SU, Kim JH (2015). Fracture patterns in the maxillofacial region: a four-year retrospective study. J Korean Assoc Oral Maxillofac Surg.

[8] Salentijn EG, Peerdeman SM, Boffano P (2014). A ten-year analysis of the traumatic maxillofacial and brain injury patient in Amsterdam: incidence and aetiology. J Cranio-Maxillo-fac Surg.

[9] Arslan ED, Solakoglu AG, Komut E (2014). Assessment of maxillofacial trauma in emergency department. World J Emerg Surg.

[10] Arabion HR, Tabrizi R, Aliabadi E (2014). A Retrospective Analysis of Maxillofacial Trauma in Shiraz, Iran: a 6-Year- Study of 768 Patients (2004-2010). J Dent.

[11] Zhou HH, Liu Q, Yang RT (2015). Traumatic head injuries in patients with maxillofacial fractures: a retrospective case-control study. Dent Traumatol.

[12] Keenan HT, Brundage SI, Thompson DC (1999). Does the face protect the brain? A case-control study of traumatic brain injury and facial fractures. Arch Surg Chic Ill 1960.

[13] Saini P (2013). Oral and maxillofacial trauma, 4th edition. Br Dent J.

[14] Goh EZ, Beech N, Johnson NR (2021). Traumatic maxillofacial and brain injuries: a systematic review. Int J Oral Maxillofac Surg.

[15] Fonseca R, Barber HD, Powers M (2012). Oral and Maxillofacial Trauma.

[16] Abosadegh MM, Rahman SAB (2018). Epidemiology and incidence of traumatic head injury associated with maxillofacial fractures: A global perspective. J Int Oral Health.

[17] Bellamy JL, Mundinger GS, Reddy SK (2013). Le Fort II fractures are associated with death: a comparison of simple and complex midface fractures. J Oral Maxillofac Surg Off.

[18] Lucke-Wold B, Pierre K, Aghili-Mehrizi S (2021). Facial Fractures: Independent Prediction of Neurosurgical Intervention. Asian J Neurosurg.

[19] Zandi M, Seyed Hoseini SR (2013). The relationship between head injury and facial trauma: a case-control study. Oral Maxillofac Surg.

[20] Goil DP, Jain DA, Gupta NK (2016). Association of Head Injury and Maxillofacial Trauma: A Prospective Case Control Study. Indian J Appl Res.

[21] Ansari MH (2004). Maxillofacial fractures in Hamedan province, Iran: a retrospective study (1987-2001). J Cranio-Maxillo-fac Surg.

[22] Kishanrao S (2021). Traumatic Brain Injury in the Elderly is common but is not as Bad as we Think! Exercise, not rest, can ensure faster recovery from post-concussion syndromes ‘Autobiographical case report. J Neurol Neurol Sci Disord.

[23] Haug RH, Adams JM, Conforti PJ (1994). Cranial fractures associated with facial fractures: a review of mechanism, type, and severity of injury. J Oral Maxillofac Surg.

[24] Louis M, Agrawal N, Kaufman M (2017). Midface Fractures I. Semin Plast Surg.

[25] Esmer E, Delank KS, Siekmann H (2016). Gesichtsverletzungen bei Polytrauma − Mit welchen Verletzungen ist zu rechnen. Notf Rettungsmedizin.

[26] Pietzka S, Kämmerer PW, Pietzka S (2020). Maxillofacial injuries in severely injured patients after road traffic accidents—a retrospective evaluation of the TraumaRegister DGU® 1993–2014. Clin Oral Investig.

